# Experimental study on direct tensile fatigue performance of basalt fiber reinforced concrete

**DOI:** 10.1038/s41598-024-51403-1

**Published:** 2024-01-08

**Authors:** Xutao Zhang, Chao Lou, Xikuan Lyu

**Affiliations:** School of Architecture and Engineering, Liaocheng University, Liaocheng, 252059 China

**Keywords:** Civil engineering, Composites

## Abstract

Conducting research on the fatigue performance of concrete materials is of great significance for the anti fatigue design of concrete structures. Currently, indirect tensile or compressive strength tests are commonly used to study the fatigue performance of basalt fiber reinforced concrete, but there is little research on its fatigue performance under direct tensile conditions. Using a fatigue testing machine and a self-developed concrete axial tensile device, direct tensile fatigue tests of basalt fiber reinforced concrete were conducted under different fiber content and stress levels. Based on fatigue test data, the entire fatigue tensile process of basalt fiber reinforced concrete was analyzed, and the effects of fiber content and stress level on the fatigue life of concrete specimens were explored. Strain fatigue life curves of concrete with different fiber content were plotted. The experimental results indicate that the failure mode of basalt fiber reinforced concrete under cyclic loading is brittle failure; with the increase of basalt fiber content, the fatigue life of concrete first increases and then decreases. When the fiber content is 0.3%, the fatigue life of basalt fiber concrete is the highest compared to the benchmark concrete. When the fiber content is the same, the fatigue life of concrete decreases with the increase of stress level. The fatigue deformation process of basalt fiber reinforced concrete can be divided into three stages: the stage of fast strain growth, the stage of uniform strain growth, and the stage of rapid strain growth.

## Introduction

During long-term service of concrete structures, in addition to bearing static forces, such as the dynamic effects of traffic loads such as cars and trains on bridges, the repeated impacts of waves on oil extraction platforms, and the effects of earthquake and wind loads on high-rise building structures^1^.This fatigue load with periodic cyclic characteristics can cause continuous changes and redistribution of internal stress fields in concrete materials, promote the continuous expansion and derivation of initial microcracks, causing progressive deterioration and fatigue damage to the mechanical properties of concrete materials, and ultimately lead to brittle failure of structural components below allowable stress^2,3^. Therefore, conducting research on the fatigue performance of concrete materials is of great significance for the anti fatigue design of concrete structures.

As a new generation of composite materials, fiber reinforced concrete has gradually become one of the widely used materials in civil engineering. By adding fiber materials such as steel fibers, carbon nanotubes^4–6^, basalt fibers, etc., the mechanical properties and durability of concrete can be significantly improved. In the past decade, domestic and foreign scholars have conducted systematic experimental research on the fatigue performance of fiber reinforced concrete, and have achieved relatively rich research results^7,8^. Among them, basalt fiber reinforced concrete is increasingly being used due to its excellent mechanical properties.

Basalt fiber is a relatively new type of inorganic fiber produced in a harmless manner through the environmentally friendly melting process of volcanic rocks^9–11^. It has excellent comprehensive properties, such as high chemical stability, low usage cost, and good compatibility with silicates, making it a good candidate material for reinforcing materials^12^. Compared with benchmark concrete, basalt fibers not only improve the strength and flexural toughness of concrete^13–16^, but also enhance the fatigue resistance of concrete.

In recent years, domestic and foreign scholars have mainly conducted the following research on basalt fiber reinforced concrete. Sim^9^ studied the mechanical and deformation properties of basalt fiber reinforced concrete with different fiber contents. Dias^17^ investigated the effect of adding different volume fractions of basalt fibers on the fracture toughness of ordinary silicate concrete through extensive experiments. The results indicate that compared to ordinary silicate concrete, the concrete with basalt fiber added has stronger fracture energy. Li^18^ studied the fatigue performance of basalt fiber reinforced polymer rod reinforced sea sand concrete beams through four point bending tests. Yang^19^ studied the evolution of fine crack structures during the service life of basalt fiber reinforced concrete under different fatigue loads, explored the influence of basalt fiber on the fatigue crack structure of concrete, and established a correlation model between flexural strength and internal crack characteristics.Guo^20^ conducted fatigue tests on basalt fiber reinforced bridge concrete using three-point bending tests, exploring the inhibitory effect of basalt fiber on concrete cracks under fatigue loads.

At present, research on the fatigue performance of basalt fiber reinforced concrete at home and abroad is mostly focused on the bending fatigue performance and compressive fatigue performance under three or four point loading, and there is little literature on its direct tensile fatigue performance. Based on this, a fatigue testing machine and a concrete axial tensile device were used to conduct direct tensile fatigue tests on basalt fiber reinforced concrete under different fiber content and stress levels. The effects of fiber content and stress level on the tensile fatigue performance of concrete were investigated, and the strain fatigue life curve was plotted, providing theoretical and experimental basis for the tensile fatigue design of basalt fiber reinforced concrete.

## Test overview

### Sample production

The design strength of basalt fiber concrete is 30 MPa, and the cement uses P.O42.5 grade ordinary portland cement, the particle size of the crushed stone is 5–20 mm, and the fineness modulus of fine aggregate is 2.5, and the grading of coarse and fine aggregates is good. The concrete mix ratio is: cement:water:sand:crushed stone = 414.89:195:608.63:1181.48, and the physical and mechanical properties of the basalt fibers used are shown in Table 1.

**Table 1:** Physical and mechanical parameters of basalt fibers.

Project	Diameter (μm)	Length (mm)	Density (kg/m^3^)	Tensile strength (MPa)	Elastic modulus (GPa)	Elongation at break (%)
Basalt fiber	15	6	2800	3000–3500	79.3–93.1	3.2

**Figure 1:**
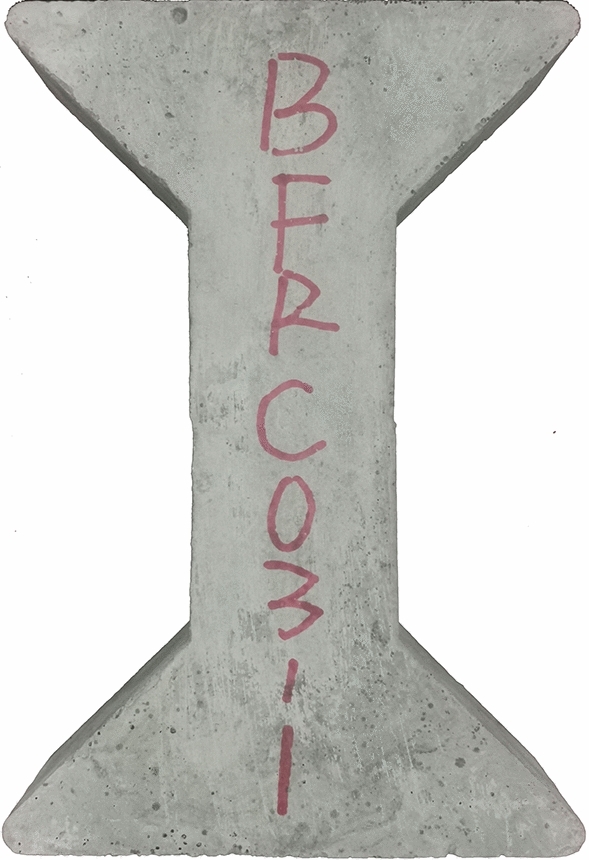
Basalt fiber reinforced concrete specimen.

Calculate and determine the amount of cement, water, sand, coarse aggregate, and basalt fiber used. Before the pouring of the sample, the coarse aggregate was first cleaned and air-dried. During the mixing process, first add coarse aggregate and sand, stirring for 60 s, then add cement and half water, stirring for 30 s, and then add basalt fibers in two more batches, stirring for 60 s.Finally, add the other half of water and stir the mixture for 180 s. Add water and basalt fibers in two steps to achieve the desired stirring state. Then measure the slump of each sample immediately after mixing. Subsequently, pour the mixed concrete into a dumbbell shaped mold and vibrate evenly on a vibration table. After pouring and demoulding, immediately move the sample to a standard concrete curing room with a constant temperature of 20 ± 2 ℃ and a relative humidity of not less than 95%. The curing time is 28 days, and care should be taken to avoid damaging the specimen during this process. The specimens formed by pouring are shown in Fig. 1.

### Test conditions

When conducting direct tensile fatigue tests on basalt fiber reinforced concrete, two main factors are considered: the amount of basalt fiber added and the stress level (the ratio of the maximum stress value of the specimen to the ultimate tensile strength under static load during fatigue testing). The length of the basalt fiber used is 6 mm, considering three different mixing amounts, they are 0.2%, 0.3%, and 0.4% respectively.Consider three different stress levels, namely 0.75, 0.8, and 0.9. Twelve dumbbell shaped specimens were made with each fiber content, of which three were used to test the static tensile strength of the specimens, and the remaining nine specimens were used for three groups of fatigue tensile tests at different stress levels, with three specimens for each set of stress levels.

### Test equipment

#### Axial tensile test device for concrete

**Figure 2:**
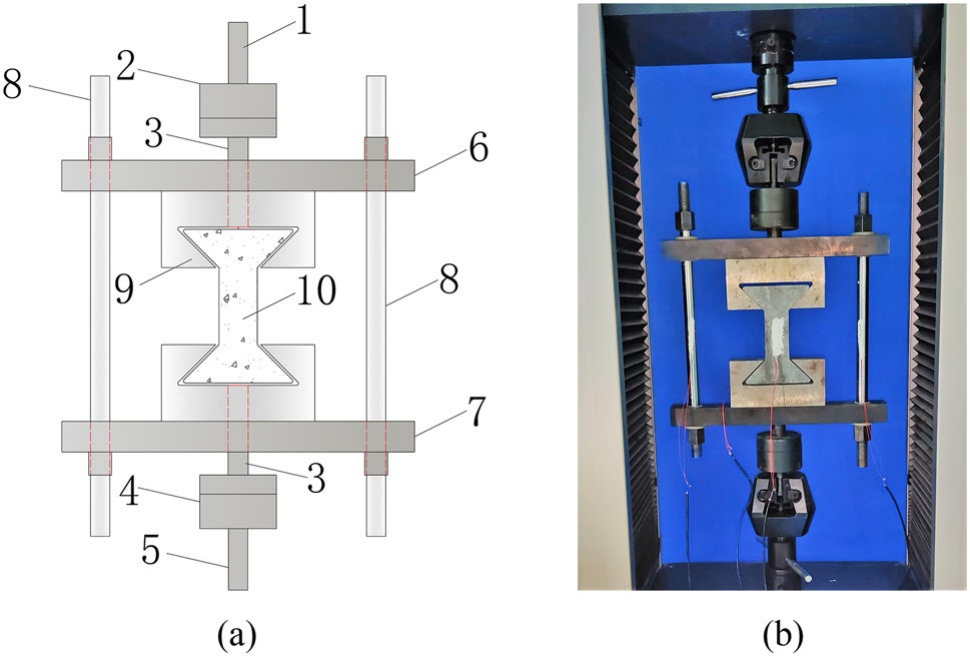
Axial tensile test device for concrete.

The axial tensile test device for concrete includes three parts: a rigid frame component, a ball joint component and a pull head, and a specimen production component, as shown in Fig. 2.

**Figure 3:**
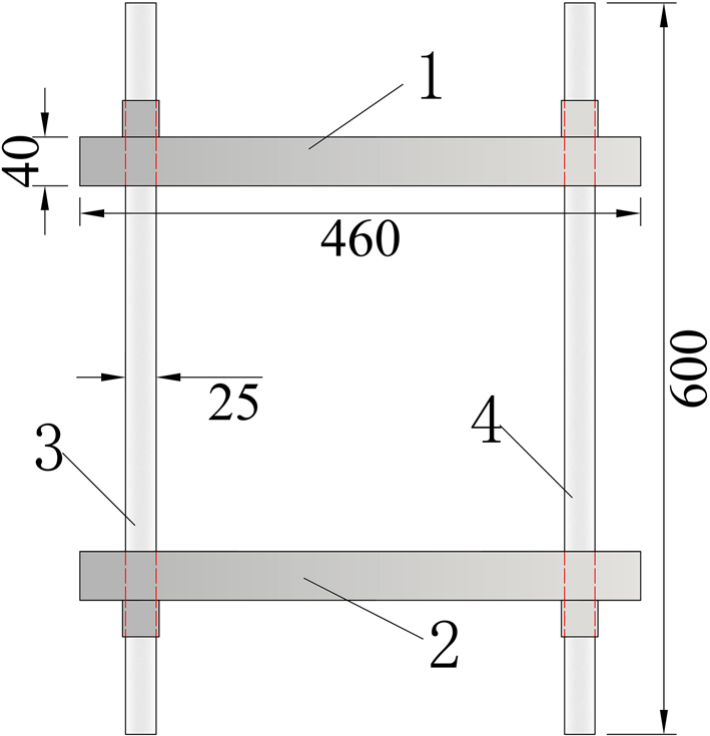
Schematic diagram of rigid frame part.

**Figure 4:**
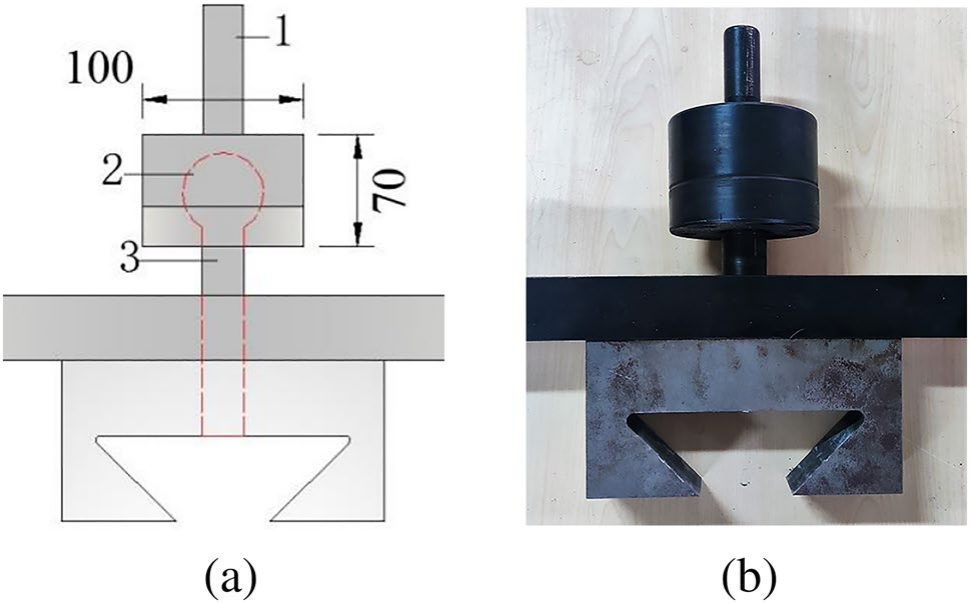
Spherical hinge part and puller.

**Figure 5:**
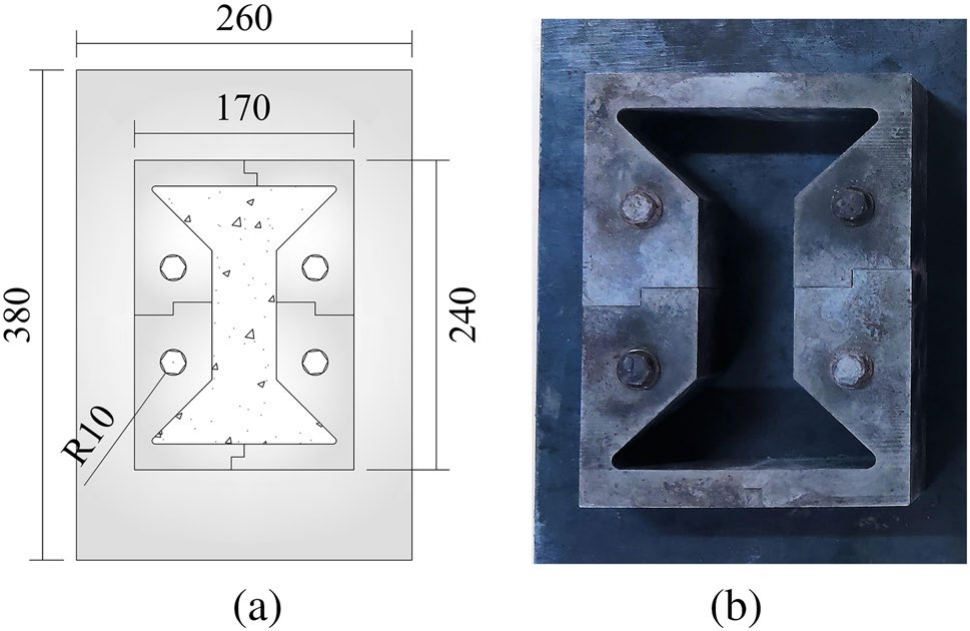
Concrete specimen mold.

Figure 3 is the schematic diagram of rigid frame part, the rigid frame consists of an upper beam, a lower beam and two tensile round steels, the round steel is connected and fixed with the beam through the nuts at the upper and lower ends; the upper and lower beams are 460 mm long, 50 mm wide and 40 mm thick; the diameter of two round steels is 25 mm and the length is 600 mm. During the test, the tensile round steel and the concrete specimen are pulled together to solve the problem that it is difficult to measure the tensile stress–strain curve after the peak strength of concrete. (2) In order to solve the eccentricity problem, the spherical hinge part and puller are designed, as shown in Fig. 4. The spherical joint is located between the concrete specimen and the pull head of the testing machine and can rotate within a certain range. During the test, it can ensure that the tensile force and the axis of the test piece are on the same axis, so as to eliminate the eccentricity problem. (3) According to the characteristics of easy placing and forming of concrete material, the author designed concrete specimen with dumbbell shape and made mould. Dimensions of dies and specimens are shown in Fig. 5. The tension zone in the middle of the specimen is 100 mm in length, 50 mm in width and 50 mm in thickness. The die consists of 5 parts, 1–4 is side die and 5 is base plate. The side die is bolted to the base plate. When making the test piece, first assemble the mold and pour the concrete, disassemble the mold after its initial setting, and take out the test piece for curing. The dumbbell shaped concrete specimen is perfectly matched with the pull head, which is not only convenient to apply tension to the specimen, but also eliminates the stress concentration caused by clamping or bonding, and effectively improves the accuracy and success rate of the test. In addition, in order to facilitate the placing of the test piece, a 3 mm gap is reserved between the puller and the test piece, and a rubber pad is set at the contact position between the puller and the test piece to eliminate the stress concentration caused by local contact.

#### Fatigue testing machine

**Figure 6:**
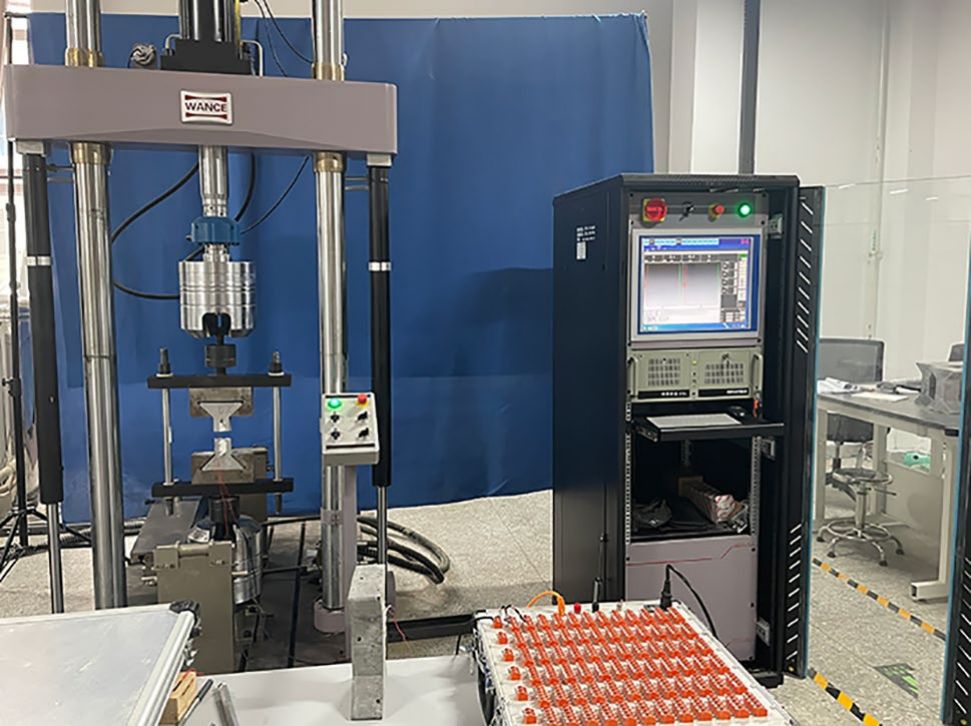
Fatigue testing machine.

**Figure 7:**
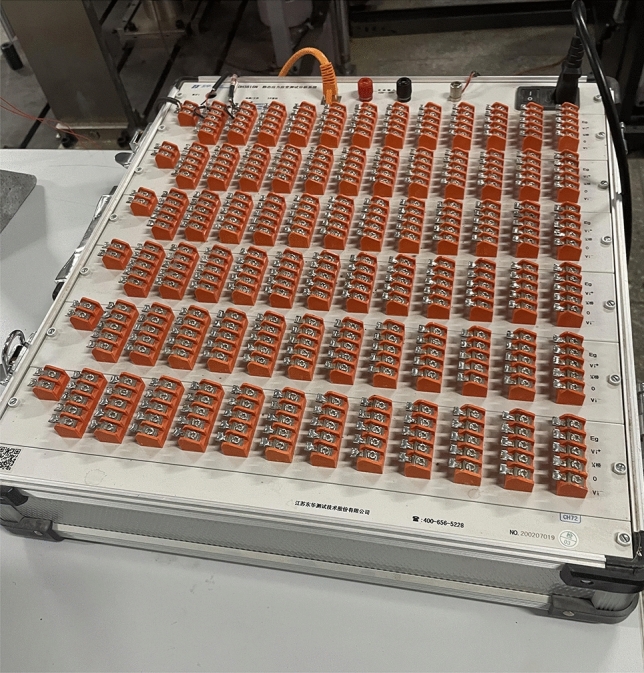
Dynamic and static strain collector.

The fatigue testing machine used in the experiment is shown in Fig. 6, which can dynamically collect cyclic load and deformation data. The testing machine can complete tests such as high cycle fatigue, low cycle fatigue, crack propagation, and fracture mechanics. The maximum load of the testing machine is 100 kN, and the loading methods include force control, displacement control, and deformation control. The highest loading frequency is 100 Hz, and the loading waveforms include sine wave, square wave, triangular wave, trapezoidal wave, oblique wave, random wave, etc. The testing machine is equipped with a dynamic and static strain gauge (as shown in Fig. 7) to collect strain data, with a maximum dynamic sampling frequency of 200 Hz. It can be connected using full bridge, half bridge, or three wire 1/4 bridge connections.

## Direct tensile static load test of basalt fiber reinforced concrete

### Test steps

The axial tensile static load test of concrete is carried out using a self-developed testing device, and the main test steps are as follows:

1. Assemble the test device. According to the schematic diagram (Fig. 2), assemble the rigid frame part, spherical hinge part and pull-down head of the concrete axial tensile device.
2. Debug the test device. The tensile space of the universal testing machine was adjusted, and the upper and lower pull bars of the device were connected and clamped with the upper and lower fixtures of the testing machine, and strain gauges were pasted on the round steels on both sides and tested with electricity.
3. Install concrete test specimen. The cured specimen is taken out and the surface is dried for the test; the specimen is installed between the pull up and down heads of the test device, and the strain gauge is pasted on the front and rear sides; open the testing machine, first apply a small tension, observe the strain of the round steel on both sides, ensure that the strain of the round steel on both sides is the same, if not, you can adjust the fastening bolts at both ends of the round steel, after debugging, the tension will be unloaded to zero.
4. The specimen is loaded. Open the testing machine, apply tension in the way of equal strain, and continue to load until the specimen is completely fractured; Record the tensile force *F* of the testing machine, the tensile strain *ε*_*0*_ of the round steel and the tensile strain *ε* of the concrete specimen, then the tensile force of the concrete specimen is:
  1 $${F}_{0}=F-{E}_{0}{\varepsilon }_{0}A$$

In the formula, *E*_0_ is the elastic modulus of the round steel, *A* is the cross-sectional area of the round steel on both sides, and *ε*_0_ is the tensile strain of the round steel.

The tensile stress of concrete specimens is:

2 $${f}_{t}=\frac{{F}_{0}}{{A}_{c}}$$

In the formula, *A*_*c*_ is the cross-sectional area in the middle of the concrete specimen.

Based on the above experimental data, the tensile strength, peak tensile strain, and complete stress–strain curve of basalt fiber reinforced concrete can be obtained.

### Direct tensile static load test results and analysis

The average static tensile strength of basalt fiber reinforced concrete measured by experiments is shown in Table 2. The numbering rules for test specimens are as follows: C represents reference concrete, BFRC represents basalt fiber concrete,the numbers 0.2, 0.3, and 0.4 after BFRC represent basalt fiber content (volume fraction) of 0.2%, 0.3%, and 0.4%, respectively.

**Table 2:** Static tensile strength of basalt fiber reinforced concrete.

Test piece type	Tensile strength (MPa)
C	1.444
BFRC0.2	1.468
BFRC0.3	1.786
BFRC0.4	1.770

**Figure 8:**
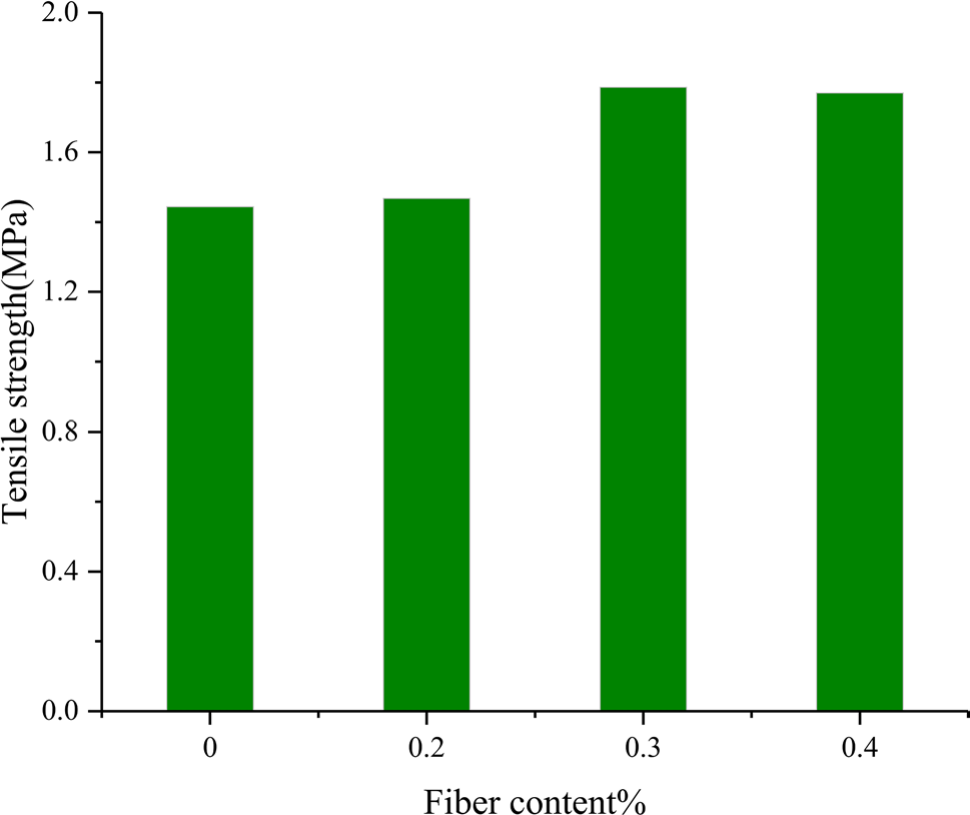
Static tensile strength of basalt fiber concrete with different fiber contents.

As shown in Fig. 8, with the increase of basalt fiber content, the tensile strength of concrete first increases and then decreases; when the volume fraction is less than 0.2%, there are too few fibers inside the concrete, making it difficult for the fibers to play a role in enhancing crack resistance. When the fiber volume fraction is 0.3%, the fibers are evenly distributed in the concrete, with more fibers distributed on the crack surface, fully utilizing the performance of the fibers and preventing the development of cracks. When the fiber volume fraction exceeds 0.3%, excessive fibers are prone to clumping inside^21,22^, which will actually reduce the tensile strength of the concrete. From the perspective of tensile strength, the optimal fiber content for basalt fiber reinforced concrete is 0.3%.

## Direct tensile fatigue test of basalt fiber reinforced concrete

### Test steps

1. After assembling the test device, install the test piece between the upper and lower heads of the test device, and stick strain gauges on both sides.
2. Before the formal start of the test, select any specimen for pre-testing, apply a load of 1 kN to the specimen, and repeat the test three times. The purpose of the pre-test is to detect whether the instrument has any problems such as poor contact or fast loading speed. After the detection is correct, input the corresponding loading frequency, average load, load amplitude, loading waveform and other test parameters, and then turn on the fatigue testing machine to load the specimen.
3. Open the fatigue testing machine, select the force controlled loading method, apply cyclic load, with a loading waveform of sine wave and a loading frequency of 10 Hz. The characteristic value of the load cycle (stress ratio) is $\rho =\frac{{P}_{min}}{{P}_{max}}=0.1$, *P*_min_ is the minimum value of constant amplitude cyclic load acting on the specimen, while* P*_max_ is the maximum value of constant amplitude cyclic load acting on the specimen, continuously loaded until the specimen completely fractures; simultaneously record the tensile strain of the concrete specimen ε and fatigue life N. If the specimen does not fail after 10^6^ cycles of loading, it is considered to have an infinite fatigue life under this loading condition.

### Direct tensile fatigue test results and analysis of basalt fiber reinforced concrete

#### Test phenomenon

**Figure 9:**
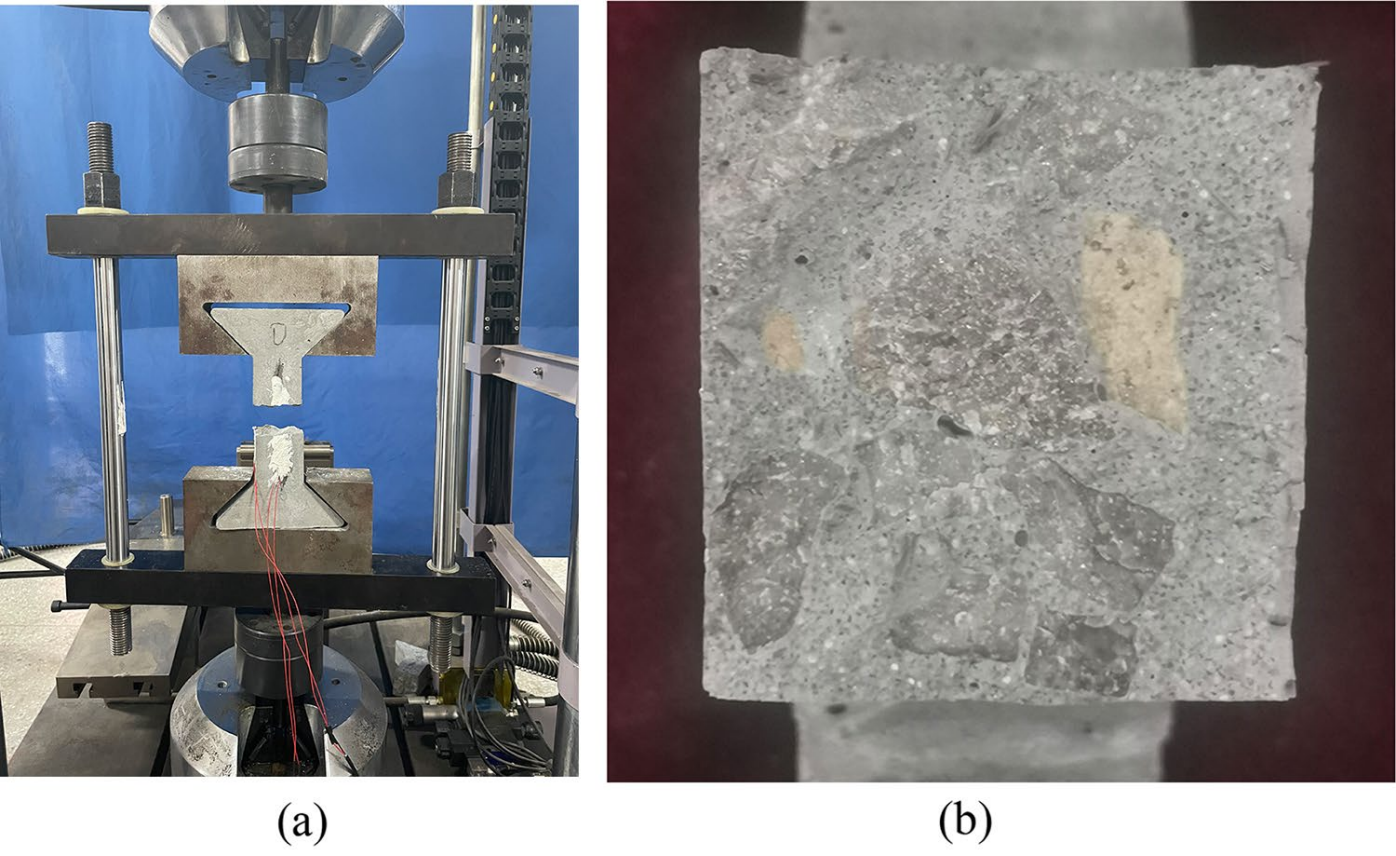
Fatigue failure morphology of basalt fiber reinforced concrete specimens under direct tension.

As shown in Fig. 9, most of the basalt fiber concrete specimens are broken along the middle position of the specimens under the circulating load. A few seconds before the failure occurred, fine cracks appear on the specimen surface, followed by fracture and failure, accompanied by a dull sound. The fracture surface of the test piece is flat, basically perpendicular to the axis of the test piece, without protruding coarse aggregate. There is no obvious damage to the concrete on both sides of the fracture surface. The main forms of fracture are tensile failure of the coarse aggregate and separation failure of the interface between the coarse aggregate and cement mortar. Basalt fibers are basically pulled out and peeled off, with very little fracture failure.

#### Test results

The fatigue life and ultimate fatigue strain of basalt fiber reinforced concrete specimens measured by experiments at various stress levels are shown in Table 3.

**Table 3:** Fatigue Life and Ultimate Fatigue Strain of Basalt Fiber Reinforced Concrete Specimens.

Test piece type	Stress level	Fatigue life (times)			Average fatigue life (times)	Ultimate fatigue strain (10^–6^)
		Test-piece 1	Test-piece 2	Test-piece 3		
C	0.9	605	767	899	757	127
	0.8	19,670	21,259	23,924	21,618	151
	0.75	39,912	49,246	72,245	53,801	176
BFRC0.2	0.9	711	936	1228	958	155
	0.8	33,524	47,812	50,621	42,986	156
	0.75	82,286	136,101	163,799	127,395	198
BFRC0.3	0.9	1098	1259	1501	1286	192
	0.8	42,715	74,700	93,030	69,148	215
	0.75	106,198	164,494	260,971	177,221	237
BFRC0.4	0.9	902	1198	1412	1171	176
	0.8	37,738	65,279	82,235	61,751	189
	0.75	94,395	143,112	218,782	152,096	213

#### The influence of fiber content and stress level on the fatigue performance of concrete

The variation pattern of concrete fatigue life with basalt fiber content is shown in Table 3. Due to the different fiber content of concrete specimens, their fatigue life shows significant dispersion at three stress levels; even though the fatigue life of three specimens in the same group still exhibits discreteness at the same stress level, which is similar to the research results of many domestic and foreign scholars. The reason for this phenomenon is, on the one hand, that the fatigue life of concrete exhibits strong sensitivity to the stress level under loading, and on the other hand, it may be due to the process issues during specimen production and the uneven distribution of various constituent materials in the concrete, resulting in differences in the strength of concrete specimens and affecting their overall structural performance, at the same time, errors in the data collection process and measurement system can also have a certain impact on the experimental results.

##### 1. Influence of fiber content on the fatigue performance of concrete

**Figure 10:**
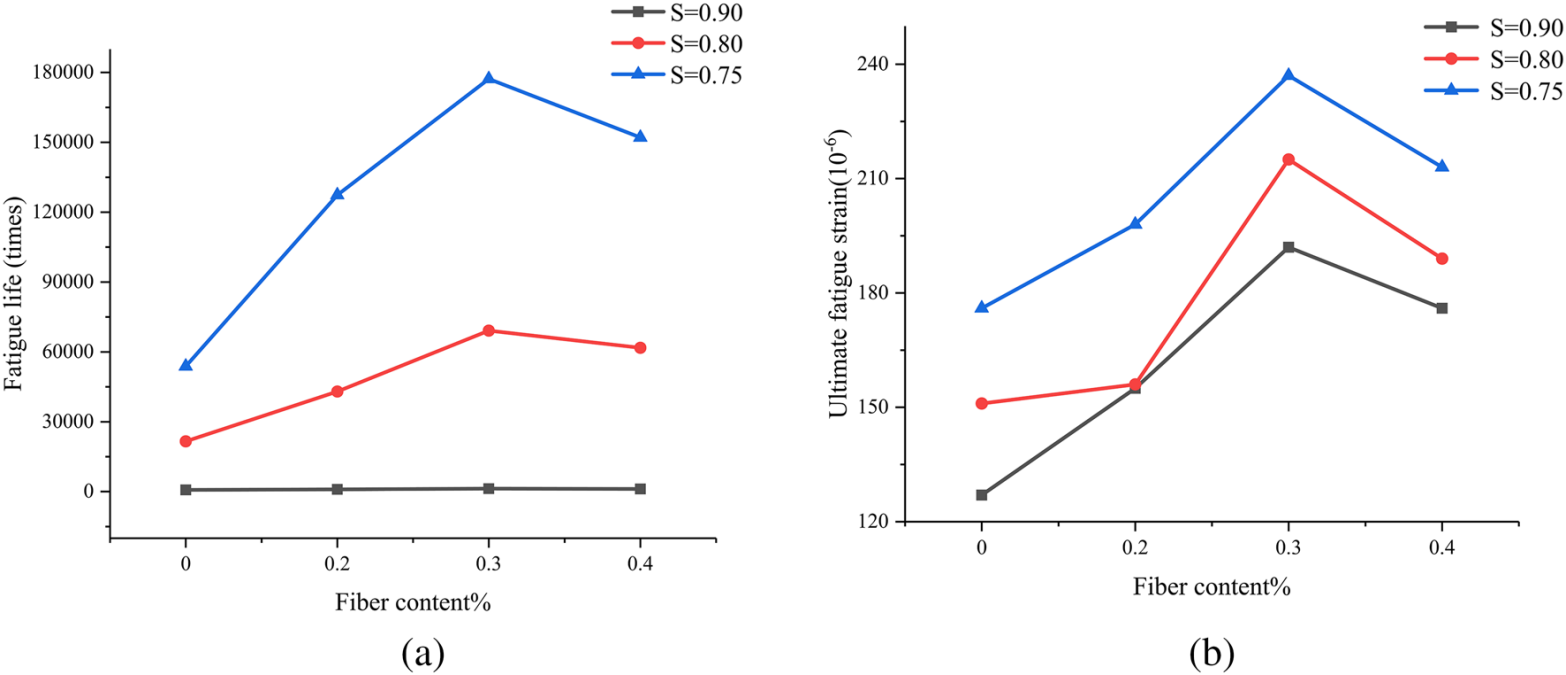
Influence of fiber content on the fatigue performance of concrete.

The variation pattern of concrete fatigue life with basalt fiber content is shown in Fig. 10a. When the stress level is the same, with the increase of basalt fiber content, the fatigue life of basalt fiber concrete first increases and then decreases, and reaches its highest at a fiber content of 0.3%. The ultimate fatigue strain of concrete also shows a pattern of first increasing and then decreasing with the increase of basalt fiber content, as shown in Fig. 10b, with the highest value at a fiber content of 0.3%. This is because the porosity inside the benchmark concrete is relatively large, and when subjected to large external forces, the pores in the matrix are easily connected to form penetrating cracks, leading to the destruction of the benchmark concrete. When basalt fibers are added, the fatigue life will increase, this is because basalt fibers can fill the pores in the concrete, and the uniformly distributed basalt fibers in the concrete can effectively bond with cement mortar, when the concrete is subjected to external forces, it can offset some of the energy, effectively hindering the generation and expansion of cracks, and overall improving the toughness and deformation resistance of the concrete, simultaneously improving the fatigue resistance of concrete. However, fibers are non directional distributed in concrete, when the fiber content is large, the agglomeration phenomenon will occur in the concrete, producing bubbles and irregular holes in the fiber overlap, which increases the probability of internal defects in the concrete, causing stress concentration inside the concrete and reducing the fatigue life of the concrete.

##### 2. Influence of stress level on the fatigue performance of concrete

**Figure 11:**
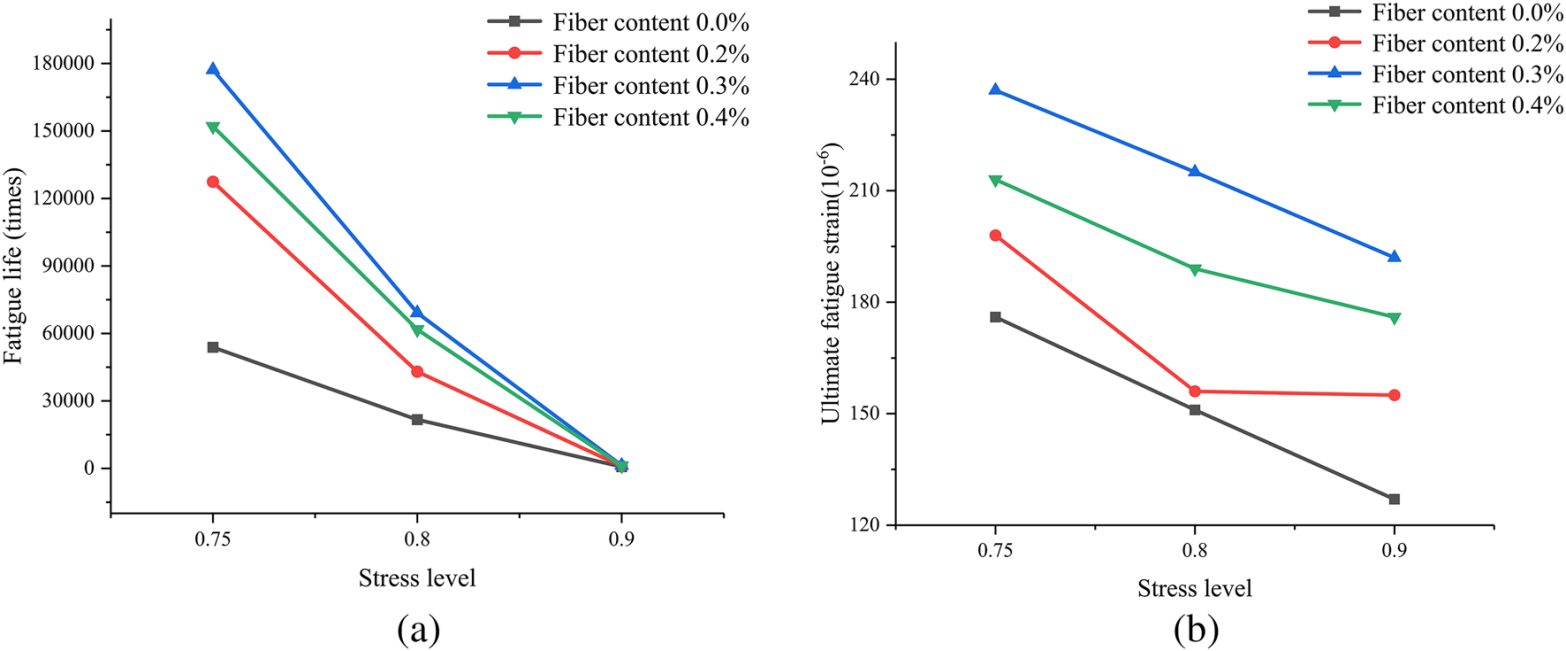
Influence of stress level on fatigue performance of concrete.

The variation pattern of concrete fatigue life with stress level is shown in Fig. 11a. When the fiber content is the same, the fatigue life of concrete specimens increases with the decrease of stress level. There are significant differences in the ultimate fatigue strain of basalt fiber reinforced concrete under different stress levels (the ultimate fatigue strain is the strain corresponding to the fatigue failure point). As shown in Fig. 11b, the lower the stress level, the greater the ultimate fatigue strain of basalt fiber reinforced concrete. The effect of stress level on the ultimate fatigue strain of basalt fiber reinforced concrete may be related to the slip amount of the fibers. When the basalt fiber concrete specimen approaches fatigue failure, the basalt fiber is in a fully activated state due to cracking and expansion. As the crack continues to expand, the basalt fibers at the main crack are continuously pulled out. In this case, a lower stress level means a smaller pulling force, which helps to increase the slip of basalt fibers. Therefore, as the stress level decreases, the ultimate fatigue strain of basalt fiber reinforced concrete increases.

#### Fatigue stress–strain curve

**Figure 12:**
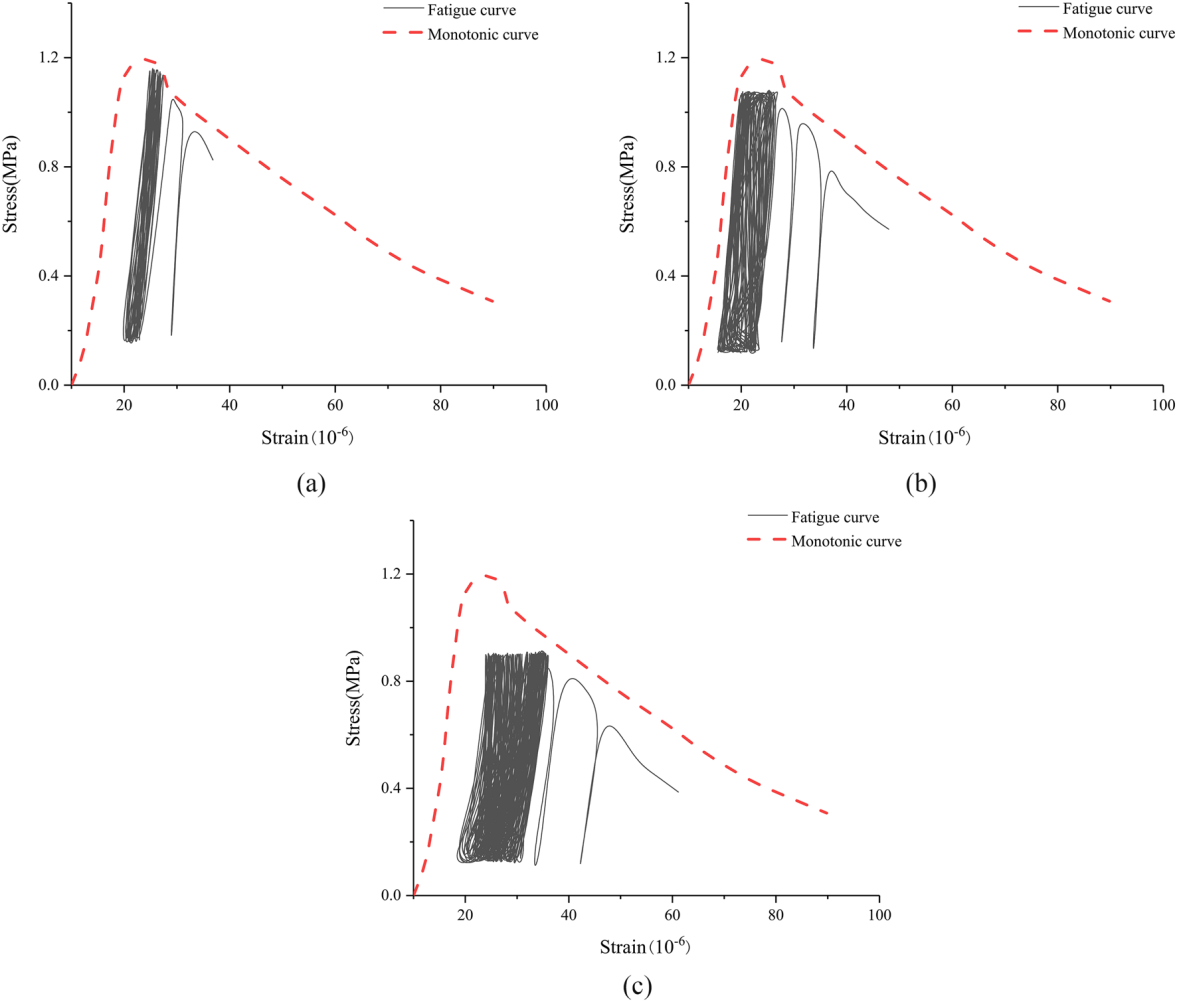
Fatigue stress–strain curve of basalt fiber reinforced concrete with a fiber content of 0.2%.

The fatigue stress–strain curves of basalt fiber reinforced concrete specimens at different stress levels are shown in Fig. 12. As the number of load cycles and plastic strain increase, the fatigue stress–strain curve gradually deviates from the monotonic curve and continues to develop towards an increase in deformation. When approaching the fatigue deformation limit, the slope of the curve decreases, reflecting the degradation of the stiffness of basalt fiber reinforced concrete specimens; the curve becomes sparse, reflecting an increase in deformation rate. When the curve begins to deviate and cannot be closed, fatigue failure occurs and cyclic loading terminates. As shown in Fig. 12, as the stress level decreases, the distance between the fatigue stress–strain curve and the monotonic curve increases, indicating that the fatigue curve gradually transitions from a "high fine" type to a "short wide" type.

#### Strain fatigue life curve

**Figure 13:**
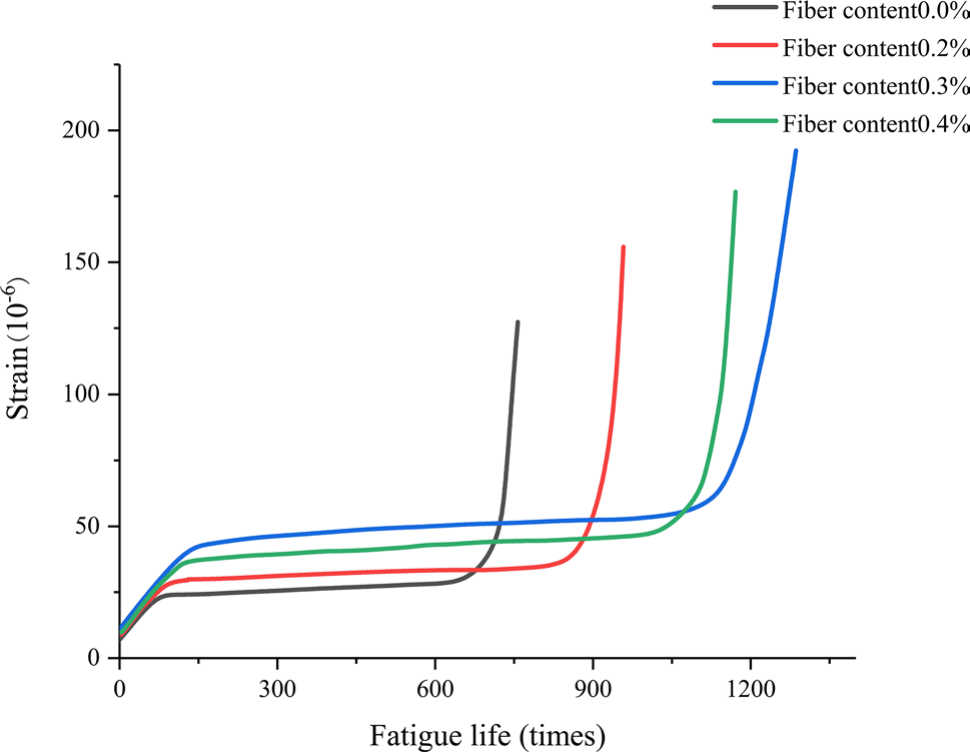
Strain fatigue life curve of basalt fiber reinforced concrete at a stress level of 0.9.

**Figure 14:**
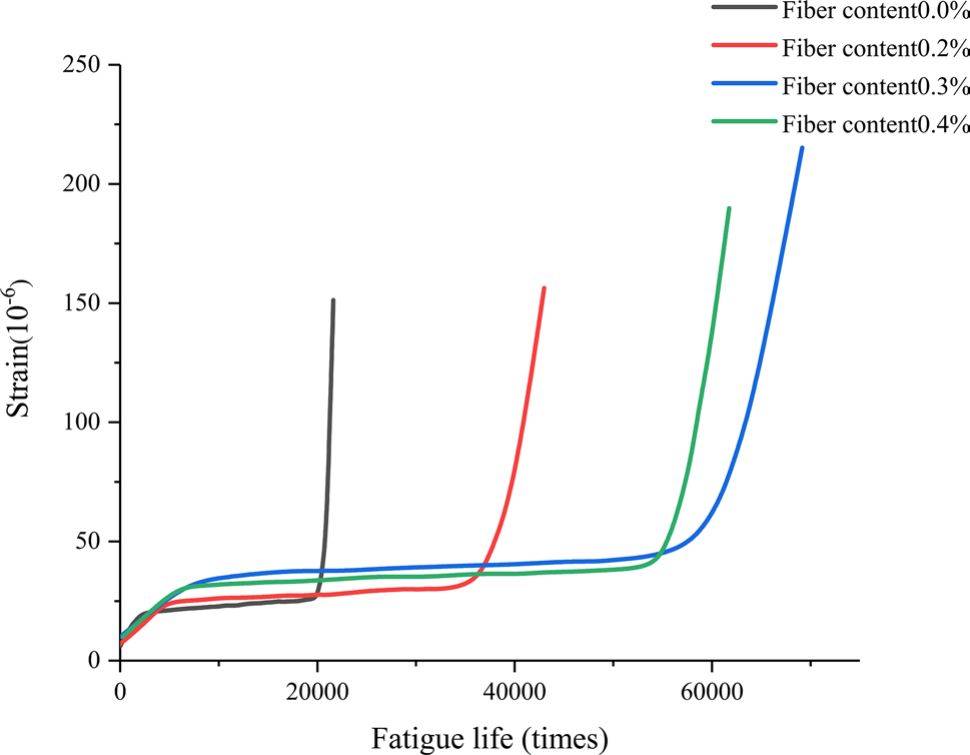
Strain fatigue life curve of basalt fiber reinforced concrete at a stress level of 0.8.

**Figure 15:**
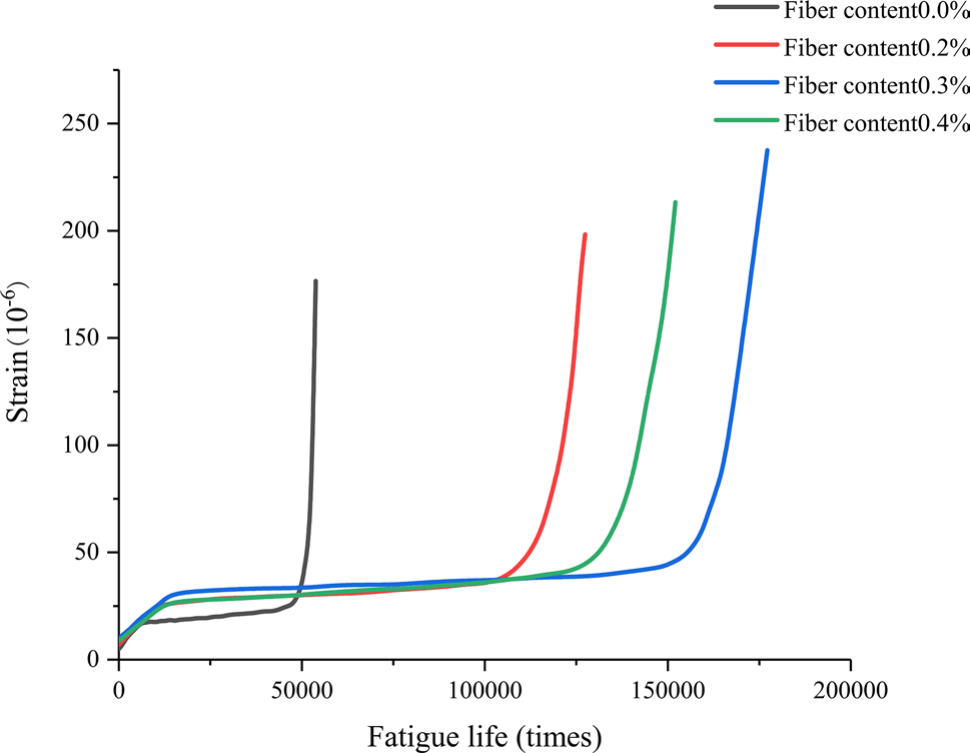
Strain fatigue life curve of basalt fiber reinforced concrete at a stress level of 0.75.

From Figs. 13, 14 and 15, it can be seen that the strain fatigue life curves of concrete specimens with different fiber contents can be divided into three stages:

In the first stage, during the initial loading stage, the strain increases rapidly, and during this stage, the initial defects inside the concrete develop rapidly under external forces. These initial defects are often small pores formed during the pouring process, and as the pores collapse and fail, the growth of strain tends to stabilize and enters the middle stage of growth. This stage is approximately 10% of the fatigue life of the specimen.

In the second stage, the middle stage of fatigue loading, in this stage, the strain growth of concrete is slow and stable, and basically develops linearly. During this period, the cracks formed in the early stages of growth extend to the surface of hard aggregates and basalt fibers in the material, which hinder crack development and bear stress. This stage experiences the longest time, which is 75% to 80% of the fatigue life of the specimen.

In the third stage, in the later stage of loading, the strain of concrete becomes steep from the stable development in the middle stage of growth, and the greater the increase at the end of its lifespan, the steeper the curve. It can be considered that a portion of the crack splits along the colloid, while a portion runs through the aggregate and a small portion of the fibers. With the increase of the material that exits the force by splitting, the remaining the remaining material bears increasing stress, causing the crack to expand more and more rapidly, ultimately forming a fracture surface that runs through the entire section, causing the specimen to fail. This stage is 10% to 15% of the fatigue life of the specimen.

Due to differences in fiber content and stress levels, there will be significant differences in the time proportion of the three stages corresponding to different concrete specimens. When the fiber content is the same, as the stress level increases, the time proportion of the first stage will correspondingly decrease. When the stress level is kept constant, compared with the base concrete, the strain fatigue life curve of basalt fiber reinforced concrete is relatively smooth, and the transition between the three stages is not very obvious. This is because the benchmark concrete has low strength, weak toughness, and due to the existing voids in the matrix, it quickly fails. After the addition of basalt fibers, the strength and toughness of concrete are greatly improved. When the specimen cracks, the basalt fibers in the matrix can play a bridging tensile role, effectively suppressing the generation and expansion of cracks. When the specimen is completely destroyed, the basalt fibers in the matrix are pulled out, offsetting a portion of the energy and greatly extending the failure time of the specimen.

## Conclusion

1. With the increase of basalt fiber content, the tensile strength of concrete first increases and then decreases; when the fiber content is 0.3%, the tensile strength of concrete is most significantly improved.
2. The fatigue failure morphology of concrete specimens with different fiber contents is basically consistent, roughly breaking into two sections from the middle; however, the fracture process of basalt fiber reinforced concrete is relatively slow compared to the benchmark concrete, and its ultimate fatigue strain is also relatively large.
3. Due to the internal non-uniformity of concrete materials, the fatigue life of concrete often has a certain discrete. When the fiber content of basalt fiber concrete is the same, the fatigue life of the material decreases with the increase of stress level. At the same stress level, the fatigue life of the benchmark concrete is the smallest, and the addition of basalt fibers effectively improves the fatigue life of the concrete; when the content of basalt fiber is 0.3%, the effect of improving the fatigue life of concrete is most significant.
4. The stress and strain of concrete during cyclic loading were tested, and the fatigue stress–strain curves of basalt fiber reinforced concrete at different stress levels were plotted. As the stress level decreases, the fatigue stress–strain curve gradually transitions from a "high fine" type to a "short wide" type.
5. The strain and fatigue life of concrete during cyclic loading were tested, and the strain fatigue life curves of concrete with different fiber contents were plotted. During the cyclic loading process, the strain process of concrete materials can be divided into three stages, namely: the stage of fast strain growth, the stage of uniform strain growth, and the stage of rapid strain growth.

## Data Availability

The authors confirm that the data supporting the findings of this study are available within the article.
